# Right ventricular outflow tract stenting for late presenter unrepaired Fallot physiology: a single-center experience

**DOI:** 10.3389/fcvm.2024.1340570

**Published:** 2024-02-01

**Authors:** Radityo Prakoso, Yovi Kurniawati, Sisca Natalia Siagian, Aditya Agita Sembiring, Damba Dwisepto Aulia Sakti, Brian Mendel, Indah Pratiwi, Olfi Lelya, Oktavia Lilyasari

**Affiliations:** ^1^ Division of Pediatric Cardiology and Congenital Heart Disease, Department of Cardiology and Vascular Medicine, National Cardiovascular Center of Harapan Kita, Universitas Indonesia, Jakarta, Indonesia; ^2^ Department of Cardiology and Vascular Medicine, Sultan Sulaiman Government Hospital, Serdang Bedagai, Indonesia; ^3^ Department of Cardiology and Vascular Medicine, National Cardiovascular Center of Harapan Kita, Universitas Indonesia, Jakarta, Indonesia

**Keywords:** adults, ejection fraction, late presenter, tetralogy of Fallot, palliative, RVOT stent

## Abstract

**Objectives:**

The purpose of this study was to assess the clinical outcome after right ventricular outflow tract (RVOT) stenting in late presenter patient with unrepaired Fallot physiology.

**Background:**

In younger patients, RVOT stenting is an alternative to mBTT shunt; however, there have been few reports of this palliative technique in late presenter population, including adults.

**Methods:**

This was a single-center, retrospective study of nonrandomized, palliated Fallot patients. Clinical outcomes such as left ventricular ejection fraction and saturation were measured in 32 individuals following RVOT stenting in adults (*n* = 10) and children (*n* = 22). The Statistical Package for Social Science (SPSS) 26.0 software was used to analyze the statistical data.

**Results:**

During the procedure, the average stent diameter and length were 8.84 ± 1.64 mm and 35.46 ± 11.23 mm, respectively. Adult patients received slightly longer stents than pediatric patients (43.60 ± 11.64 mm vs. 31.77 ± 9.07 mm). Overall, patients' saturation increased from 58.56 ± 19.03% to 91.03 ± 8.98% (*p* < 0.001), as did their left ventricular ejection fraction (LVEF) from 64.00 ± 18.21% to 75.09 ± 12.98% (*p* = 0.001). Three patients improved their LVEF from 31 to 55%, 31 to 67%, and 26 to 50%. The median length of stay was 8 (2–35) days, with an ICU stay of 2 (0–30) days. The median time from RVOT stent palliation to total repair was 3 months (range: 1 month–12 months).

**Conclusions:**

RVOT stenting is a safe and effective method for increasing saturation and ejection fraction not only in newborn infants but also in late presenters, including adults with unrepaired Fallot physiology.

## 1 Introduction

The initial palliative strategy of patients with ventricular shunts and narrow right ventricular outflow tract (RVOT) with reduced pulmonary blood flow remains challenging. During the last decades, creation of modified Blalock–Thomas–Taussig (mBTT) shunt has been well established, however complication and mortality has yet to be very high (1, 2). To countermeasure this issue, right ventricular outflow tract (RVOT) stenting has been developed as initial palliation of symptomatic patients. However, the efficacy of RVOT stenting is only highlighted in current literature for newborns and young children (3, 4).

Late presentations in developing countries is common, making the problems more complicated. Previously, uncorrected Fallot physiology in late presenter patients was exclusively treated conservatively with medication, especially with significant desaturation and diminished ventricular function. Tetralogy of Fallot patients frequently showed late in low-income countries, accounting for 85.1% of cases (5, 6).

Nonetheless, there have been few reports of the benefit of RVOT stenting in patients with unrepaired Fallot physiology, particularly in late presenter patients (7, 8). Therefore, this institutional experience aims to investigate the outcome of RVOT stenting in late presenter patients with Fallot physiology.

## 2 Methods

### 2.1 Study design and setting

Between December 2019 and April 2023, thirty-two patients with unrepaired ventricular shunts and severely narrow right ventricular outflow tract physiology resulting in profound desaturation were considered for RVOT stenting in a retrospective, single-center study. The local hospital ethics review board approved RVOT stenting as an alternative to mBTT shunt. The primary endpoints were saturation and ejection fraction before and after palliation in late presenter tetralogy of Fallot patients (beyond one year old). Subjects were chosen after a multidisciplinary team meeting in a weekly surgical conference. The decision not to operate is justified by evidence-based medicine and in the patient's best interest considering very high mortality in this patient's group. The main indications for RVOT stenting in late presenters and adults in our center are (1) Profound desaturation (typically below 40%–50%), (2) Low ejection fraction (LVEF <40%), (3) Patient who had a high risk for performing mBTT shunt, and (4) Patient who had a high risk for total repair despite adequate pulmonary artery size (4, 5).

Each procedure were performed while the patients were sedated and mechanically ventilated. To enable for biplane angiography, patients were positioned on the table with their arms lifted. To prevent hypothermia, all patients were given a warming device. A sterile dressing was applied to the patient's chest and upper abdomen to enable for intraprocedural echocardiographic assessment with a standard ultrasonography probe in a sterile sleeve. The preferred method was right femoral or right internal jugular venous access, while in certain circumstances a carotid approach may be employed (9).

The first right ventricular angiography was conducted with a diagnostic catheter positioned near the apex of the right ventricle with a 30″ RAO, 30″ cranial tilt, and a straight lateral projection. Heparin 50 IU per kgBW was administered to the patient. The diagnostic catheter was removed from the right ventricle and inserted in the superior vena cava following the angiography. The right ventricular outflow tract, pulmonary valve annulus, and branch pulmonary arteries were measured and compared to earlier or simultaneous ultrasonography data (9).

### 2.2 Stent choice

The size and type of stent were chosen based on Kirklin's full size and the anticipated length of palliation. Our center had stent lengths of 28 mm, 39 mm, and 56 mm available. The type and diameter of the stent were chosen in general based on the patient's body weight, the length of the RVOT infundibulum, and the need for palliative surgery. We employed peripheral vascular stents in adult and adolescents patients, and coronary stents in young children above the age of one year. The stent's diameter is typically 1–2 mm bigger than the measured infundibulum during diastole, and the stent's length should include the distal muscular section of the RVOT, the pulmonary valve, and/or the main branch of the pulmonary artery before the bifurcation (9).

Following stent selection, the suitable delivery sheath or guide catheter was chosen. A 4–5 F Right Judkins catheter was pushed past the tip of the delivery sheath after the delivery sheath was inserted in the superior vena cava. On the hub of the Judkins catheter, a spinning haemostatic valve was attached to the pressure line and side arm contrast syringe. A 0.014″ coronary wire was threaded through the haemostatic valve near the catheter's tip. After withdrawing the entire system, the right ventricle was reintroduced under pressure monitoring and the right ventricular outflow tract was intubated using the catheter. Instead of the wire, side arm test injections were utilized to check the location of the catheter. The catheter was then moved to the distal branch pulmonary artery while continuous pressure was recorded and an angiography was performed. The coronary wire was inserted into the distal branch pulmonary artery, and the delivery sheath or guide catheter was advanced into the distal branch pulmonary artery over the diagnostic catheter. The diagnostic catheter was then withdrawn from the coronary wire, and the sheath was aspirated and flushed (9).

The wire was wrapped around the pre-mounted stent, which was then advanced to the desired spot within the RVOT. The stent was slightly exposed, and side arm test injections were repeated. When the position was deemed satisfactory, the stent was totally exposed. The delivery balloon was manually inflated with one hand while the stent system was managed with the other. Following stent implantation, the balloon was gradually deflated as the delivery sheath was advanced over the balloon to re-sheath it. This enabled for a secure placement of the delivery system's tip into the stent (9). Predilatation with a coronary or vascular balloon should be considered if necessary, especially in cases of multilayer stenosis. Following the procedure, furosemide IV 1 mg/kgBW IV and milrinone drip 0.375 mg/kgBW IV were administered. A repeat angiography and a brief ultrasound assessment were performed. The balloon was subsequently advanced over the pulmonary valve in all cases for valvuloplasty. Oxygen saturations were regularly measured.

Another cardiac ultrasonography assessment and right ventricular angiography were performed. An additional blood gas study was performed. Finally, the coronary wire was removed under fluoroscopic control through the delivery sheath, the delivery sheath was removed, and manual hemostasis was applied. Hemoglobin should be 14g% after the procedure.

### 2.3 Statistical presentations

Exploratory analysis with graphical and tabular displays evaluated evidence in favor of trends and associations. Normally distributed data was presented as mean ± standard deviation. Data that is skewed is presented as the median, minimum, and maximum. Where appropriate, categorical data is expressed as counts and percentages. Boxplot graphs depicted the difference in saturation and ejection percent between the pre- and post-palliation procedures. The Shapiro–Wilk test was used to determine the normality of numerical data because the subjects were less than 50. The paired *t*-test was used to compare the change in both saturation and LVEF before and after the procedure. The Statistical Package for Social Science (SPSS) 26.0 software was used for all data analyses (10). *p* < 0.05 values were considered significant.

## 3 Results

**Figure 1:**
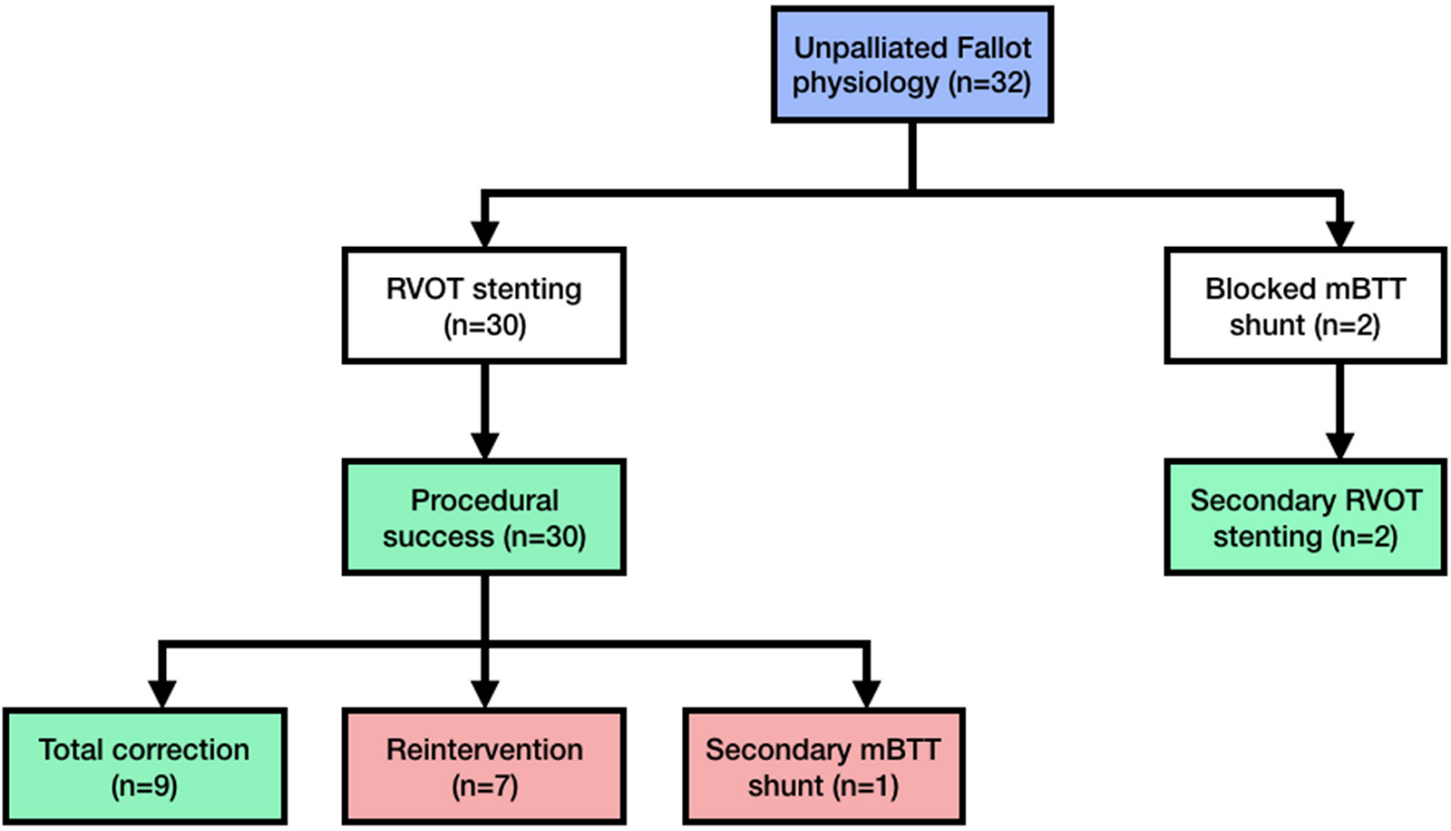
Patient flow diagram showing the cohort of unrepaired late presenter fallot physiology. Every patient who received RVOT stenting was included in this diagram. This study did not record any intraprocedural patients deaths. mBTT modified-Blalock–Thomas–Taussig; RVOT right ventricular outflow tract.

The RVOT stenting was performed on 32 individuals, with the patient flow diagram depicted in Figure 1. Table 1 shows the baseline parameters of this investigation. In two cases, mBTT shunt was performed initially; however, due to blocked mBTT shunt, the patients were scheduled for rescue (secondary) RVOT stenting (rescue RVOT stent was performed to improve the patient clinical condition). The patient was followed for a median of 13.5 (1–31) months. There were 14 female patients (43.75%). The median age at stent implantation was 9.5 (1–40) years. The median weight ranged from 23 (5.5–55) kilos. During presentation, ten adult patients (31.25%) had unpalliated Fallot physiology. Nine were diagnosed with tetralogy of Fallot.

**Table 1:** Patient characteristics.

Demographics	
Patients (*n*, %)	32 (100%)
Female (*n*, %)	14 (43.75%)
Weight at initial palliation, kg	23 (5.5–55)
Age at initial palliation, years	9.5 (1–40)
Underlying anatomy and comorbidities	
Tetralogy of Fallot	26 (81.25%)
DORV, VSD, PS	3 (9.37%)
Inlet muscular VSD, PS	1 (3.12%)
Tricuspid atresia, ASD, VSD, PS	2 (6.25%)

ASD, atrial septal defect; DORV, double outlet right ventricle; PS, pulmonary stenosis; VSD, ventricular septal defect.

### 3.1 Procedural details

**Figure 2:**
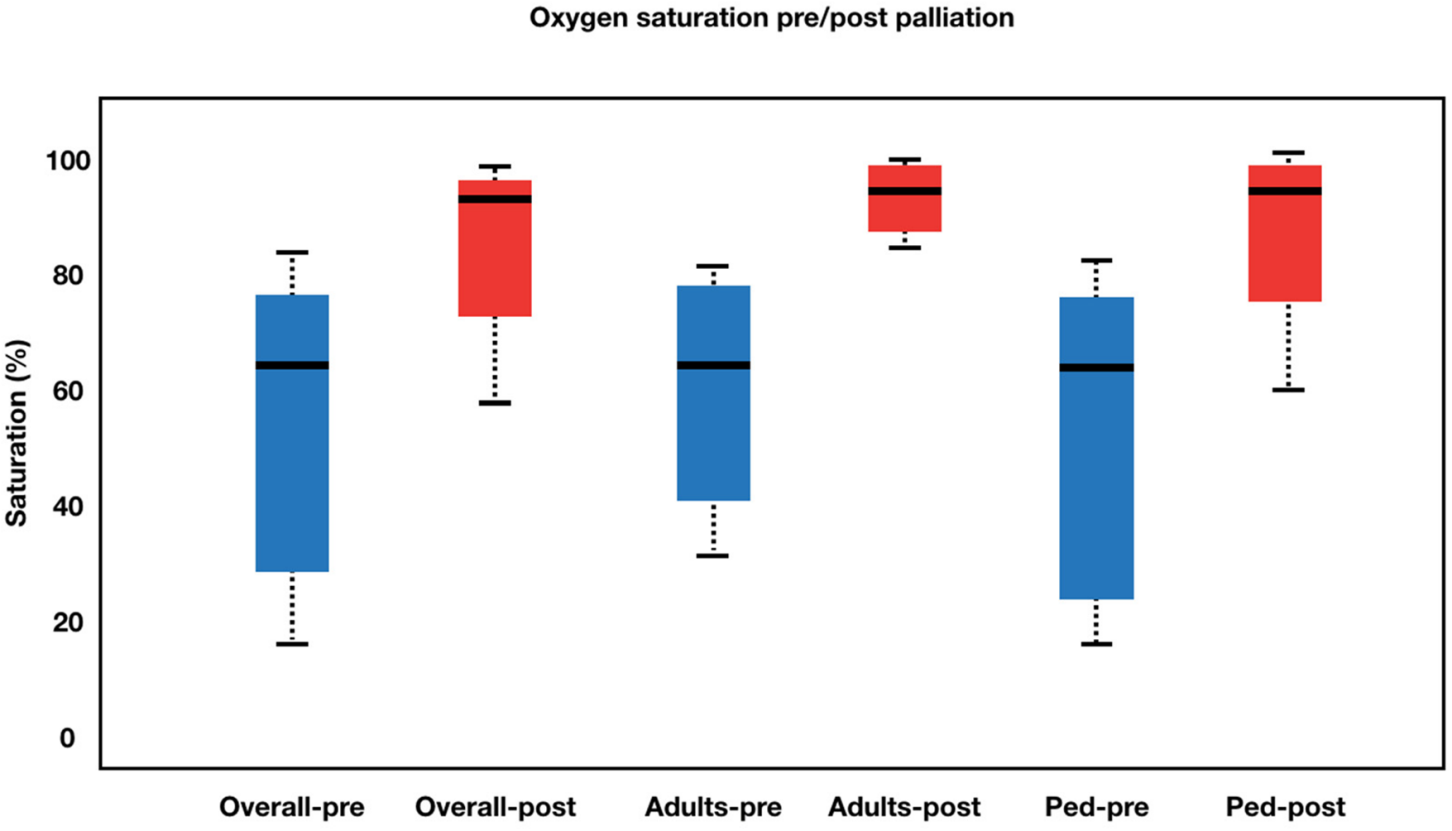
Boxplot diagram illustrating oxygen saturation pre- and post-palliation. Overall, patients’ saturation went from 58.56 ± 19.03% to 91.03 ± 8.98% (*p* < 0.001). Adults’ saturation went from 65.20 ± 15.36 to 92.00 ± 5.29 (*p* = 0.005), while pediatrics’ saturation went from 55.54 ± 20.08 to 90.59 ± 10.32 (*p* < 0.001). Pre-palliation is shown in blue. Post-palliation is shown in red. Box is first to third quartile. Median is thick line in box. The whiskers extend to the data point that is no more than 1.5 times the interquatile range from the box.

**Figure 3:**
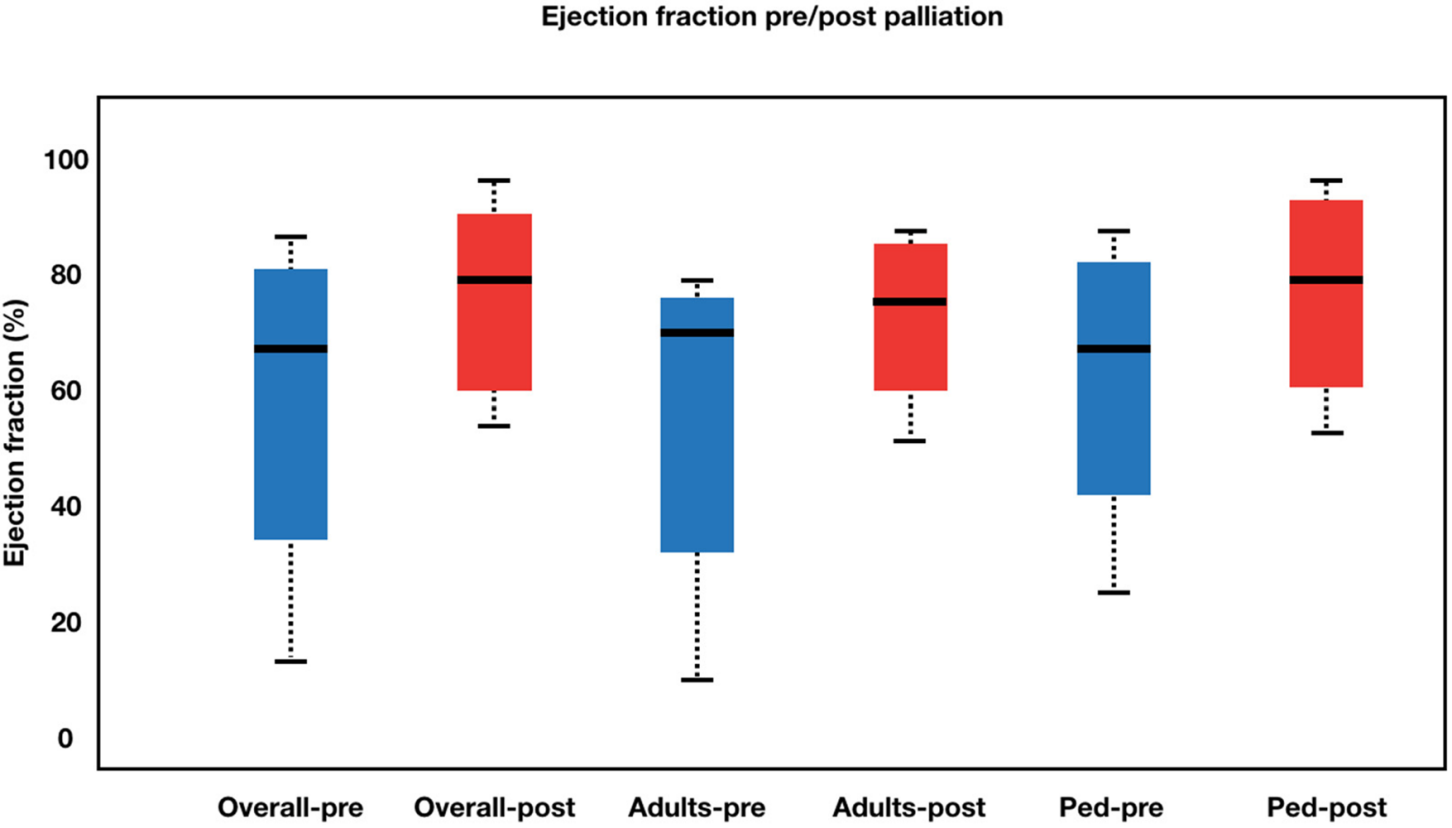
Boxplot diagram illustrating ejection fraction pre- and post-palliation. The overall left ventricular ejection fraction (LVEF) increased from 64.00 ± 18.21% to 75.09 ± 12.98% (*p* = 0.001) with adults’ LVEF went from 60.33 ± 19.37 to 70.66 ± 12.13 (*p* = 0.097) and pediatrics’ LVEF saturation went from 65.50 ± 17.96 to 76.90 ± 13.15 (*p* = 0.002).Pre-palliation is shown in blue. Post-palliation is shown in red. Box is first to third quartile. Median is thick line in box. The whiskers extend to the data point that is no more than 1.5 times the interquatile range from the box.

The procedural details was summarized in Table 2. The approach for this procedure was 26 femoral, 5 jugular and 1 carotid. All adults patient was performed using the femoral approach. Median fluoroscopy time was 17.9 (6.07–44.6) minutes. During the learning curve stage, the procedure time was greatly reduced. During the procedure, the average stent diameter and length were 8.84 1.64 mm and 35.46 ± 11.23 mm, respectively. Adult patients received slightly longer stents than pediatric patients (43.60 ± 11.64 mm vs. 31.77 ± 9.07 mm). Overall, patients' saturation went from 58.56 ± 19.03% to 91.03 ± 8.98% (*p* < 0.001). Adults' saturation went from 65.20 ± 15.36 to 92.00 ± 5.29 (*p* = 0.005), while pediatrics' saturation went from 55.54 ± 20.08 to 90.59 ± 10.32 (*p* < 0.001). The overall left ventricular ejection fraction (LVEF) increased from 64.00 ± 18.21% to 75.09 ± 12.98% (*p* = 0.001) with adults' LVEF went from 60.33 ± 19.37 to 70.66 ± 12.13 (*p* = 0.097) and pediatrics' LVEF saturation went from 65.50 ± 17.96 to 76.90 ± 13.15 (*p* = 0.002). Figures 2, 3 illustrate boxplots of the saturation and ejection fractions comparing adult and pediatric populations. Three patients improved their LVEF from 31 to 55%, 31 to 67%, and 26 to 50%. The median length of stay was 8 (2–35) days, with an ICU stay of 2 (0–30) days.

**Table 2:** Right ventricular outflow tract stenting procedural details.

Procedural variable	
Approach	
Femoral (*n*, %)	26 (81.25%)
Jugular (*n*, %)	5 (15.62%)
Carotid (*n*, %)	1 (3.12%)
Fluoroscopy time (mins)	17.9 (6.07–44.6)
Stent	
Diameter (mm)	8.84 ± 1.64
Length (mm)	35.46 ± 11.23
Pre-procedural saturation, %	58.56 ± 19.03
Post-procedural saturation, %	91.03 ± 8.98
Pre-procedural LVEF, %	64.00 ± 18.21
Post-procedural LVEF, %	75.09 ± 12.98
Length of stay, days	8 (2–35)
ICU stay, days	2 (0–30)
Complications	
Fractured stent (*n*, %)	1 (3.12%)
Dislodged stent (*n*, %)	3 (9.37%)
Reinterventions (*n*, %)	4 (12.50%)
Arrhythmias (*n*, %)	2 (6.25%)
Death (*n*, %)	3 (9.37%)
Time from initial palliation to total repair (median, min–max)	3 months (1 month–12 months)
Total repair (*n*, %)	9 (28.12%)

ICU, intensive care unit; LVEF, left ventricular ejection fraction.

### 3.2 Complications

**Figure 4:**
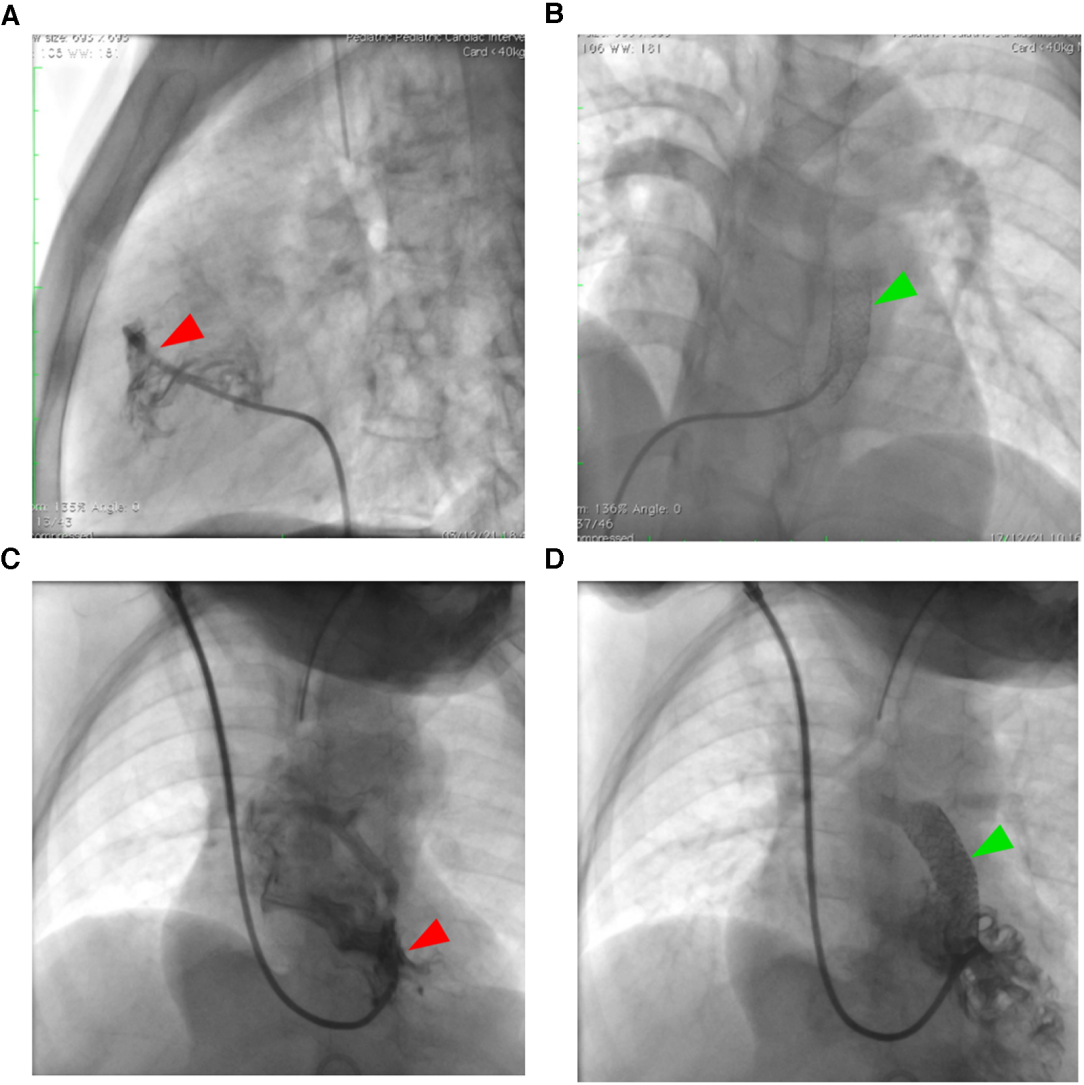
Right ventricular outflow tract stenting in unpalliated Fallot physiology. Adult patients (**A,B**) with tetralogy of Fallot face particular difficulties with RVOT stenting in comparison to pediatric population (**C,D**) because of their greater muscular infundibular pulmonary stenosis (*red arrowhead*), which increases the risk of stent fracture as seen in figure (**B**) The *green arrowhead* points to the stent.

One fractured stent was discovered in an 18-year-old woman who had reintervention using the stent-in-stent approach (see Figure 4). The patient clinical condition improved and was able to undergo total repair one week after the second RVOT stent. Three patients (9.37%) experienced a dislodged stent (two in the right atrium and one in the descending aorta), requiring a rescue RVOT stent. In three cases of dislodged stent, total repair was performed not directly after the complication, but several months after the initial palliation with the median time from RVOT stent palliation to total repair 3 months (range: 1 month–12 months). Referral to surgical ward was only to remove the dislodged stent. Arrhythmias were found in two young patients, one with supraventricular tachycardia and the other with severe bradycardia.

In both the adult and pediatric populations, there was no intraprocedural death. There was no post-procedural death in the adult Fallot physiology populations. However, three pediatric patients died from sepsis during in-hospital treatment. Total repair was performed on nine patients, four adults and five children.

## 4 Discussions

### 4.1 Right ventricular outflow tract stenting in Fallot physiology patients

Total repair was the definitive treatment for TOF patients, which was usually done before the age of one year (11). However, not all TOF patients may benefit from total repair because the remainder required palliative care for issues such as cyanotic spells, severe and broad RVOT blockage, stenotic or hypoplastic branch pulmonary artery, or ventricular dysfunction. Similarly, patients with ventricular shunts and pulmonary stenosis physiology or a blocked mBTT shunt may benefit from RVOT stenting prior to definitive treatment, as in our instance (12–14). Quandt et al. discovered that 26.6% of TOF patients required palliative care before undergoing total repair. The mBTT shunt was one of the most commonly performed palliative procedures, with a death rate ranging from 2.3 to 18.4% (4). The mBTT shunt produced various unintended consequences, including pulmonary artery deformation and stenosis, as well as the existence of cicatrix formation, which could complicate the total repair surgery (1). RVOT stents were known to have a better prognosis than mBTT shunts (2). Luxford et al. shown in their study that high-risk infants undergoing RVOT stent had lower morbidity and mortality than those undergoing mBTT shunt, and achieved an effective bridge to definitive surgery (15). However, we did not directly compare the efficacy of the procedures between mBTT shunt and RVOT stent in this study because of the different nature of the patient undergoing those two procedures in our center (This pilot study primarily included late presenter and adult patients, whereas mBBT shunts were typically performed in neonates in our center).

TOF patients commonly presented late in nations with lower incomes, accounting for 85.1% of cases. Due to limited resources, TOF is frequently identified late in adulthood. Despite advancements in diagnostic technologies and a better understanding of the ailment compared to earlier centuries, certain delayed presentations are still being documented (5, 6). Unfortunately, no additional late presenter Fallot type lesion has been studied. In our particular instance, ten patients with Fallot physiology presented as adults. Because Fallot physiology in adults is uncommon, they were initially treated with different diagnoses. Furthermore, there are no acknowledged recommendations or consensus on the age of late-presenter TOF patients or how to manage them (16–19). The majority of late-presenter TOF patients who lived into adulthood had chronic polycythemia with hyperviscosity and consumptive coagulopathy with varying manifestations. Though uncorrected TOF survival is uncommon, it has been observed that approximately 10% of affected individuals live to adulthood, with only 5% reaching the age of 40 (5, 6). Chronic hypoxia and fibrosis are thought to be the causes of late-presenter TOF patients' left or right ventricular dysfunction. Pre-operative elevated hematocrit (>45%), age greater than 4 years, and RVEDP ratio greater than 12 mmHg were identified as risk factors for cardiomyocyte degeneration and interstitial fibrosis in RVOT, which could result in ventricular dysfunction (20, 21). According to Chowdury et al., 29.3% of TOF patients aged 4–15 years and 81.8% of those aged >15 years had right ventricular dysfunction and low cardiac output (20).

### 4.2 RVOT stenting as a potential primary palliative treatment for unrepaired adults Fallot physiology

Previously, most RVOT stenting research focused on the effects of saturation and pulmonary artery growth in babies (22–26). However, in this investigation, we discovered an intriguing occurrence in which saturation and ejection fraction improved dramatically, particularly in adults with unrepaired Fallot physiology. The decision not to operate is justified by evidence-based medicine and in the patient's best interest considering very high mortality in this patient's group. The stage approach with initial palliation should be the preferential approach. Palliative non-surgical treatments may thus be considered in individuals with unrepaired Fallot physiology, cyanosis, and ventricular dysfunction, with the goal of improving ventricular dysfunction before to total repair. Li et al. demonstrated that pre-operative ventricular function predicts improved post-operative ventricular function (27). The palliative technique also served to prepare the left ventricle for enhanced blood flow when total repair was performed.

The placement of an RVOT stent is intended to enhance pulmonary blood flow, which will increase systemic saturation and possibly repair ventricular function in late-presenter TOF patients. Prolonged hypoxia in people with unrepaired Fallot physiology and borderline ventricular function resulted in hybernating myocardium. As a result, when the hybernating myocardial regained blood supply, the EF improved considerably. In contrast to pediatric populations, children had high EF despite being severely cyanotic due to their still well-maintained reserve (8).

Arrhythmia, cerebrovascular accident, tamponade, stent thrombosis, malposition and embolisation, and vascular access problems such as hemorrhage and thrombosis have previously been documented following RVOT stent insertion. Post-procedural problems were confined to a transitory conduction disruption, clinically inconsequential RVOT perforation, and proximal stent embolisation (22–26). We also discovered various issues in adult populations, such as fractured or dislodged stents, which we believe are caused by hypertrophied infundibular stenosis. However, reintervention resolves the majority of these issues (8). Interestingly, all of our adult patients improved clinically and were able to undergo total repair with no intra- or post-procedural death reported.

### 4.3 Long-term outcome: stent fracture and restenosis

Regarding RVOT stenting in late presenter tetralogy of Fallot, there were not many long-term data available, particularly for adult populations. Main causes of reduced patency with RVOT stenting include intimal hyperplasia, early thrombosis, and stent fractures. In the RVOT, fractures have been identified as a major risk factor for late stent failure. The stent was exposed to distortive stresses during the heart contractions due to the presence of very tight infundibular stenosis and hyperdynamic motion, which are common in patients who present with TOF late (7, 8). Roughly 1%–8% of cases are reported to have a stent fracture; higher prevalence is noted in structures with excessive motion and hinge movement (28). The stent's torsion and muscle compression in the RVOT context may be comparable to those in the femoropopliteal artery. Untreated stent fractures may increase the risk of in-stent restenosis and stent thrombosis (7, 8).

Total repair should ideally be carried out three to six months following initial palliation. Because of this reason, the stent was not intended to be durable for long-term use. Thus, by (1) developing stents to better respond to the dynamic mechanics of the RVOT region and (2) determining the ideal implantation site to decrease mechanical stresses, the rate of stent fracture and its related problems could be reduced.

## 5 Limitations

The limitations of this study stem from its retrospective approach and limited cohort size. The cohort is a high-risk, diverse collection of patients with distinct anatomical substrates. Because our institution's practice has evolved over time, a consistent technique was not used on all patients.

## 6 Conclusions

RVOT stenting is a safe and effective method for increasing saturation and left ventricular ejection fraction in late presenter patients with unrepaired Fallot physiology. In a few cases, repeat RVOT stenting is required prior to definitive correction of very tight pulmonary stenosis, this method allows for somatic growth and pulmonary artery rehabilitation before to definitive total repair.

## Data Availability

The original contributions presented in the study are included in the article/Supplementary Material, further inquiries can be directed to the corresponding author.
